# High water-use efficiency and growth contribute to success of non-native *Erodium cicutarium* in a Sonoran Desert winter annual community

**DOI:** 10.1093/conphys/cou006

**Published:** 2014-03-05

**Authors:** Sarah Kimball, Jennifer R. Gremer, Greg A. Barron-Gafford, Amy L. Angert, Travis E. Huxman, D. Lawrence Venable

**Affiliations:** 1 Center for Environmental Biology, University of California, Irvine, Irvine, CA 92697-1450, USA; 2 EEB, University of Arizona, Tucson, AZ 85721, USA; 3 Biosphere 2, University of Arizona, Tucson, AZ 85721, USA; 4 Department of Zoology, University of British Columbia, Vancouver, British Columbia V6T 1Z4, Canada; 5 EEB, University of California, Irvine, Irvine, CA, USA

**Keywords:** community structure, competition, enemy release hypothesis, invasive species, trade-offs, winter annual plants

## Abstract

The success of non-native, invasive species may be due to release from natural enemies, superior competitive abilities, or both. In the Sonoran Desert, *Erodium cicutarium* has increased in abundance over the last 30 years. While native species in this flora exhibit a strong among-species trade-off between relative growth rate and water-use efficiency, *E. cicutarium* seems to have a higher relative growth rate for its water-use efficiency value relative to the pattern across native species. This novel trait combination could provide the non-native species with a competitive advantage in this water-limited environment. To test the hypothesis that *E. cicutarium* is able to achieve high growth rates due to release from native herbivores, we compared the effects of herbivory on *E. cicutarium* and its native congener, *Erodium texanum*. We also compared these two species across a range of environmental conditions, both in a common garden and in two distinct seasons in the field, using growth analysis, isotopic compositions and leaf-level gas exchange. Additionally, we compared the competitive abilities of the two *Erodium* species in a greenhouse experiment. We found no evidence of herbivory to either species. Physiological measurements in a common environment revealed that *E. cicutarium* was able to achieve high growth rates while simultaneously controlling leaf-level water loss. Non-native *E. cicutarium* responded to favourable conditions in the field with greater specific leaf area and leaf area ratio than native *E. texanum*. The non-native *Erodium* was a stronger competitor than its native congener in a greenhouse competition experiment. The ability to maintain relatively higher values of water-use efficiency:relative growth rate in comparison to the native flora may be what enables *E. cictarium* to outcompete native species in both wet and dry years, resulting in an increase in abundance in the highly variable Sonoran Desert.

## Abstract (precis)

Erodium cicutarium, an invasive plant, has recently increased in abundance in the Sonoran Desert. We tested hypotheses for its success, and found no evidence for a release from natural enemies. Instead, E. cicutarium was able to achieve higher growth rates while controlling leaf-level water loss, allowing it to out-compete natives.

## Introduction

One primary goal in invasion biology is to understand how non-native species are able to outcompete native species and increase in abundance in their introduced range (Levine *et al.*, 2003; Bossdorf *et al.*, 2005; Richardson and Pysek, 2006; Moles *et al.*, 2012). The enemy release hypothesis (ERH) states that non-native species perform better in their introduced range because they experience a decrease in natural enemies that restrict performance (Keane and Crawley, 2002). It is also possible that invasive species are successful because they possess specific life history or physiological traits that allow them to outcompete natives (Rejmanek, 1996; Rejmanek and Richardson, 1996; Kolar and Lodge, 2001; Sakai *et al.*, 2001). Many physiological studies have demonstrated that invasive species have traits that enable them to grow quickly, including higher specific leaf area (SLA), leaf nitrogen content and rates of CO_2_ assimilation relative to native species (Baruch and Goldstein, 1999; Leishman *et al.*, 2007; Feng and Fu, 2008; Feng *et al.*, 2008). Thus, non-native species may be successful because they are physiologically superior to native species in terms of their ability to capture and use resources (Funk and Vitousek, 2007). These traits that frequently characterize non-natives often trade off with the ability to tolerate predation, so a release from natural enemies may allow populations with such traits to succeed in new environments.

According to the ERH, the release from natural herbivores allows non-native species to grow faster than native species that may be experiencing herbivory (ERH; Keane and Crawley, 2002). This hypothesis requires that defense mechanisms come at a cost to growth and fitness (Inbar *et al.*, 2001; Stamp, 2003). Release from herbivory could allow individuals to invest in traits for growth and resource acquisition without the associated cost, allowing them to grow larger in high-resource conditions due to a breakdown of the growth–defense trade-off (Blumenthal, 2006). If defense mechanisms result in a fitness cost, a release from herbivory could favour individuals that invest less in defense and more in growth and reproduction (Handley *et al.*, 2008). Given that competitive ability is often linked with size and fecundity (Goldberg and Fleetwood, 1987; Gurevitch *et al.*, 1990; Wang *et al.*, 2010), this release from predation could allow non-native populations to evolve to be stronger competitors (Blossey and Notzold, 1995). However, traits related to high growth rates and competitive abilities also trade off with traits related to abiotic stress tolerance, dispersal abilities and other tolerances and preferences, such that breakdowns in any of these trade-offs may explain the success of invasive species without a release from natural enemies (Turnbull *et al.*, 1999; Agrawal *et al.*, 2010; Molina-Montenegro *et al.*, 2012).

At the University of Arizona's Desert Laboratory in the Sonoran Desert, we have identified a trade-off between leaf-level water-use efficiency (WUE) and relative growth rate (RGR) in the winter annual plant community, such that species with high RGR have low WUE and vice versa (Angert *et al.*, 2007, 2009; Huxman *et al.*, 2008; Kimball *et al.*, 2013). These patterns were documented using stable isotopes of carbon (as a proxy for WUE) and growth analysis and, subsequently, have been supported by a large number of studies (Huxman *et al.*, 2013). When compared with the native flora, the non-native species present in our community (*Erodium cicutarium* and *Schismus barbatus*) have higher WUE for their given RGR (Fig. S1). The high values of RGR relative to WUE for these two non-native species may indicate an unstable community, in which the non-natives will outcompete natives (Kimball *et al.*, 2013). Our long-term demographic data indicate that one of these species, *E. cicutarium*, has experienced a slight increase in abundance over the last 30 years despite a general decline in the numbers of all winter annual species (Kimball *et al.*, 2010; Venable and Kimball, 2013). It is unclear how or why the non-natives in our system are able to achieve higher RGR for their given WUE, but an understanding of this may be critical to understanding both the spread of invasive species and the future dynamics of invaded plant communities.

*Erodium cicutarium* is native to the Mediterranean region and was introduced to the Sonoran Desert during the 1870s (Mensing and Byrne, 1998). It now occurs throughout North America and is abundant in many different habitats, including California Grassland, Mojave Desert and Chihuahuan Desert (Brooks, 2002; Kimball and Schiffman, 2003; Schutzenhofer and Valone, 2006). The competitive ability of *E. cicutarium* is known to increase with addition of nitrogen, burning, and increased precipitation in the Mojave Desert (Brooks, 2000, 2002, 2003) and with burning and cattle grazing in California grasslands (Meyer and Schiffman, 1999; Kimball and Schiffman, 2003). *Erodium cicutarium* germinated and reproduced earlier than other Sonoran Desert winter annuals over the last 30 years (Kimball *et al.*, 2011), which may help to explain its success in dry years (Kimball *et al.*, 2012).

In this study, we focused on mechanisms that may have determined the success of *E. cicutarium* in our Sonoran Desert winter annual system. We compared the performance of *E. cicutarium* with a commonly found native congener with a similar growth form, *Erodium texanum.* We chose to focus on *E. cicutarium* rather than *S. barbatus*, the other non-native species that exhibits high RGR and WUE, because *S. barbatus* is a grass, and native grasses do not occur in high abundance at our study site. In addition to being members of the same genus, *E. texanum* and *E. cicutarium* are similar to each other in many other ways, including buffered population dynamics, early life history transitions, high integrated WUE, high germination fractions and increased ability to photosynthesize at low temperatures (Venable, 2007; Huxman *et al.*, 2008; Kimball *et al.*, 2011; Gremer *et al.*, 2012). These similarities make *E. texanum* a good choice for a native comparison to clarify mechanisms driving the success of *E. cicutarium*. We also focused our attention on trait relationships of *E. cicutarium* compared with the general patterns found in the native flora. We addressed the following questions. (i) Does non-native *E. cicutarium* experience less herbivory than native *E. texanum*, as would be predicted by the ERH? (ii) Are there unique combinations of physiological traits related to use of the primary limiting resource, water, and related to growth components that enable *E. cicutarium* to achieve both high RGR and WUE? (iii) Is *E. cicutarium* able to outcompete *E. texanum*? We used a combination of manipulative field experiments, common garden contrasts and observations in natural field settings to understand the mechanisms by which *E. cicutarium*, a problematic invasive species, has succeeded in its introduced range.

## Materials and methods

### Herbivore exclusion

Herbivore exclusion plots were established at the University of Arizona's Desert Laboratory at Tumamoc Hill in Tucson, AZ, USA to test the enemy release hypothesis. Specifically, we compared the performance of the non-native, invasive species *E. cicutarium* with its native congener, *E. texanum*, in control and herbivore-exclusion plots.

On 10 December 2007, shortly after winter germination, 16 control and 16 exclusion plots were placed in areas where both species were present. We did not manipulate the density of plants in this experiment, so individuals are likely to have experienced some competition for resources in all plots. The plots were 50 cm ×50 cm, with data collected from a 25 cm × 25 cm area in the centre, and plots were placed in blocks to control for any non-visible environmental gradient. Hardware cloth was placed around each plot, with bird netting placed over the top. Carbaryl insecticide (active ingredient 1-naphthyl *N*-methylcarbamate, brand SEVIN) was applied weekly with a backpack sprayer at a concentration of 59.1 ml/3.785 L water/92.9 m^2^ (2 fl oz/gal water/1000 ft^2^) throughout the growing season. Control plots had hardware cloth placed on the south and west sides to control for the influence of shading while still allowing herbivores to enter the plot, and were sprayed weekly with water to control for any influence of additional water received during pesticide application. The amount of shading and water added to both the pesticide and control plots was minimal, and the plots did not appear any greener than the surrounding landscape, so it is unlikely that shading or watering influenced plants in the study plots.

On 1 April 2008, at the end of the growing season, all *E. cicutarium* and *E. texanum* individuals were harvested from each plot. We noted the number of individuals per species per plot, and we counted the number of fruits on each individual plant. Overall biomass was determined by weighing dried plants. To determine whether the number of individuals, the average number of fruits produced by individuals per plot, or the average biomass of individuals per plot varied depending on the block, species, or treatment, we performed separate mixed-model ANOVAs for each dependent variable, with block as a random factor.

### Physiology and growth

Our previous physiological measurements in the Sonoran Desert winter annual community indicated that *E. cicutarium* had higher RGR for its given integrated WUE (measured as carbon isotope ratios) than native species, including the congener *E. texanum* (Fig. S1). Given that integrated water-use efficiency is an estimate of instantaneous WUE over the lifetime of the leaf (Farquhar *et al.*, 1989), more detailed measures of water loss were necessary to understand whether *E. cicutarium* achieved high WUE through low conductance and/or through high values of carbon assimilation. To determine whether the native and non-native *Erodium* species have differences in their physiology that may explain differences in WUE and RGR, we grew both species in a controlled environment and conducted measurements of growth and water loss.

In late January 2009, seeds of non-native *E. cicutarium* and native *E. texanum* were germinated on agar in Petri dishes. When seedlings were 2 weeks old, 24 individuals of each species were transplanted into 164 ml ConeTainer pots (Stuewe & Sons, Inc., Corvallis, OR, USA) filled with a 2:3 mixture of 30-grit silica sand to Sunshine Soil Mix #3. Plants were placed in a single growth chamber set to a daytime high temperature of 21°C, and a night-time low of 5°C, which is close to average for the typical germination months of the winter growing season. After 2 weeks, on 4 February 2009, plants were transferred to the Desert Biome at the University of Arizona's Biosphere 2 facility (daytime high temperature of 19°C and low of 5°C). On 25 February, after all physiological measurements were completed but prior to reproduction, plants were divided into above- and below-ground material and dried at 60°C for 2 weeks to determine total biomass and the ratio of root mass to total biomass (RMR). General linear models were used to determine whether biomass varied depending on the species.

Just prior to reproduction, we measured stomatal conductance (*g*_s_) with a Decagon leaf porometer on five individuals of each species at four different times throughout the day (08.30, 10.30, 13:30 and 16:30 h). As only four measurements were taken during the day, stomatal conductance was assumed to be zero at dawn and dusk to integrate under curves with six total time points. To estimate total daily patterns of leaf water exchange with the atmosphere, the trapz function in R was used to evaluate the area under the curve during a 12 h period (Borchers, 2013). To test whether differences between species in daily water loss characteristics were due to chance, we conducted a permutation test. For each of 1000 permutations, species identities were randomized and daily water loss was estimated as described above. These randomized values were compared with observed values to test whether observed species differences were larger than expected by chance.

Using plants grown in natural settings in the field, we performed sequential harvests in 2 years with different rainfall patterns (2004–05 and 2007–08) for both *E. cicutarium* and *E. texanum* to understand how *E. cicutarium* achieved higher growth rates than natives for its given WUE. Summary growth components (e.g. SLA, RMR) during 2004–05 for *E. texanum*, but not *E. cicutarium* (despite its inclusion in the 2004–05 study), were previously reported along with other native members of the winter annual community (Angert *et al.*, 2007). We repeated this growth analysis during the 2007–08 growing season following the methods presented by Angert *et al.* (2007) to determine whether growth of the two species varied depending on environmental conditions that differed between years. We used mixed-model ANCOVAs to analyse RGR (ln-transformed biomass over time). The analysis included ln-transformed biomass as the dependent variable, year (2004–05 vs. 2007–08 growing seasons) and species (*E. cicutarium* vs. *E. texanum*) as categorical variables, and time since germination (plant age) as a covariate. Of key interest is the year-by-species interaction included in the analyses to understand the potential differential performance of the invasive vs. native species across different environmental conditions. The plot from which individuals were collected was included as a random factor. We also tested whether the rate of dry mass increase per unit area (net assimilation rate; NAR) varied depending on age, year, species, or the interaction between year and species, with a similar motivation for interpretation as RGR. Values for RGR and NAR were calculated by linear regression as the slope of ln-transformed biomass through time and ln-transformed mass per leaf area through time.

To determine whether components of RGR varied with species, year, or their interaction, we analysed SLA (the ratio of leaf area to dry leaf mass), leaf area ratio (LAR; the ratio of leaf dry mass to total plant dry mass), root:shoot ratio, or leaf mass ratio (LMR; the ratio of leaf dry mass to total plant dry mass) using mixed-model ANOVAs of ln-transformed mid-season values (just prior to reproduction, 95 days after germination in each year). Plot was included as a random factor. We also compared the mid-season relative change in LAR (LAR_95 days after germination_ – LAR_53 days after germination_/LAR_53 days after germination_) to determine how species responded to mid-season precipitation in each year. This analysis provided a means to evaluate these two species in terms of the established growth strategies identified by Angert *et al.* (2007), where species in the community were generally divided into those that responded to resource availability by increasing carbon assimilation during cool post-rain conditions (high-WUE species) vs. those that produced large amounts of leaves later in the growing season (high-LAR species). Samples of dried leaf tissue from five individuals of each species in each year were sent to the stable isotope laboratory at University of Arizona for analysis of leaf nitrogen content and carbon isotope ratios. Carbon isotope ratio values were converted to ^13^C discrimination values, or Δ (Farquhar *et al.*, 1989). Leaf nitrogen and Δ were analysed by ANOVAs with year, species and the species-by-year interaction as factors.

### Competition

Individuals of non-native *E. cicutarium* and native *E. texanum* were transplanted from the field (the Desert Laboratory at Tumamoc Hill) into pots shortly after germination on 5 and 6 February 2009 and placed in the greenhouse at the University of Arizona. Six different combinations were planted in 4-inch pots containing a 2:3 mixture of 30-grit silica sand to Sunshine Soil Mix #3. The six planting combinations were as follows: (i) *E. cicutarium* planted alone; (ii) *E. texanum* planted alone; (iii) *E. cicutarium* with four individuals of *E. cicutarium*; (iv) *E. cicutarium* with four individuals of *E. texanum*; (v) *E. texanum* with four individuals of *E. texanum*; and (vi) *E. texanum* with four individuals of *E. cicutarium*. The greenhouse was set to mimic the average daily outdoor temperatures during February and March, which ranged from 20 to 24°C. Each pot received equal amounts of water (∼6 ml in each pot) every day during the initial establishment period and every other day after the first 2 weeks. When plants had finished reproducing at the end of the growing season on 31 March 2009, we recorded the number of seeds produced by each focal plant. Plants were harvested, divided into above-ground and below-ground biomass, dried to a constant mass and weighed.

To determine the strength of competition (or facilitation) between *Erodium* species, we calculated the relative interaction intensity (RII), as follows:

$$\hbox{RII} = \frac{B_{{\rm w}} - \overline{B_{{\rm o}}}}{B_{{\rm w}} + \overline{B_{{\rm o}}}}$$

where *B*_w_ is the metric of performance with competition and $\overline{B_{{\rm o}}}$ is the mean of the metric without competition (Armas *et al.*, 2004). The average value of 20 individuals of each species grown alone ($\overline{B_{{\rm o}}}$), was calculated for each species and measurement variable, so that we could calculate RII for seed set, total biomass, above-ground mass and root mass. Using this metric, negative values of RII indicate competition, while positive values indicate facilitation. To determine whether the species interaction differed depending on the focal species, the identity of the competitor or an interaction between the two factors, we analysed the four RII values by two-way ANOVA, with species and competitor as fixed factors.

## Results

### Herbivore exclusion

**Figure 1:**
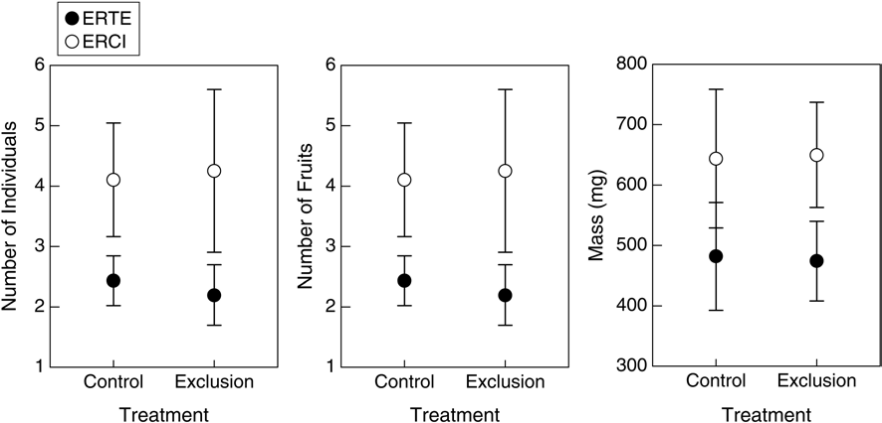
Number of individuals, mean number of fruits and mean mass of *Erodium texanum* (ERTE) and *Erodium cicutarium* (ERCI) individuals in control and herbivore exclusion plots. Values are means ± SEM.

Neither species of *Erodium* was impacted by herbivory (Fig. 1). This was true for the number of individuals (treatment *F*_1,44_ < 0.001, *P* = 0.973), for the average number of fruits (treatment *F*_1,44_ = 0.45, *P* = 0.506) and for average plant mass (treatment *F*_1,44_ < 0.001, *P* = 0.944). There were more individuals of *E. cicutarium* than *E. texanum* in all plots (species *F*_1,44_ = 4.70, *P* = 0.036), and individuals appeared to be well spaced, with a relatively low density of 376 individuals/m^2^ for all winter annual plants during the 2007–08 growing season (http://www.eebweb.arizona.edu/faculty/venable/LTREB/). The average number of fruits produced by each *E. cicutarium* individual was greater than those produced by *E. texanum* individuals (species *F*_1,44_ = 13.14, *P* < 0.001). The mass of the *E. cicutarium* individuals was significantly greater than that of individuals of *E. texanum* (species *F*_1,44_ = 4.39, *P* = 0.042). There was no evidence for the ERH, as would be detected in a species-by-treatment interaction (*P* > 0.8 in all cases).

### Physiology and growth

**Figure 2:**
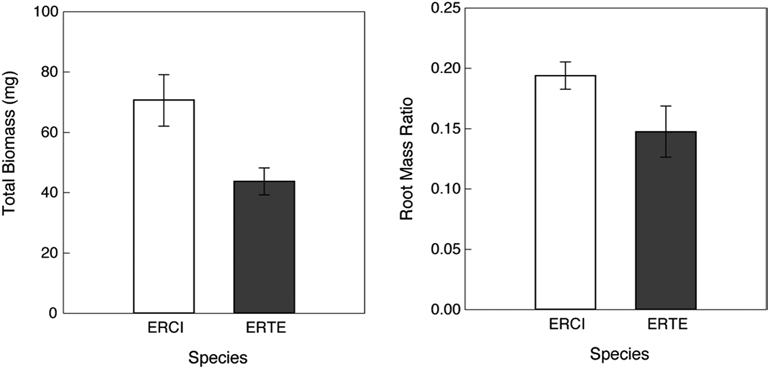
Biomass and root mass ratio (root mass/total biomass) of *E. cicutarium* (ERCI) and *E. texanum* (ERTE) grown in a common environment at the University of Arizona's Biosphere 2 facility in Tucson, AZ, USA. Values are means ± 1 SEM.

**Figure 3:**
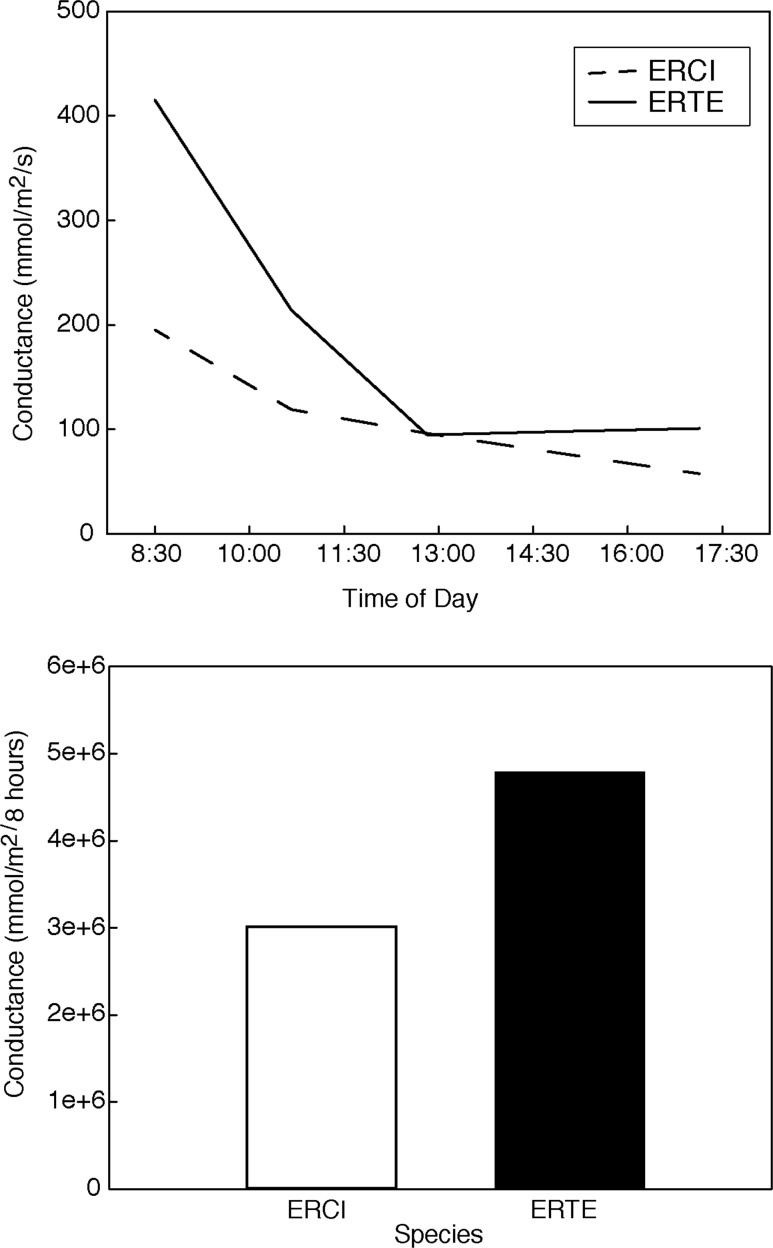
Instantaneous conductance (top panel) measured with a leaf porometer at four different times during the day on non-native *E. cicutarium* (ERCI) and native *E. texanum* (ERTE) individuals growing in pots at University of Arizona's Biosphere 2 facility. The graph below shows integrated water loss over the course of a day for the two species as estimated from instantaneous measures, with the addition of two zero values at dawn and dusk.

Individuals of *E. cicutarium* were larger than those of *E. texanum* grown over the same time period in the same environment at Biosphere 2 (Fig. 2; *F*_1,22_ = 8.21, *P* = 0.009). Non-native *E. cicutarium* had greater above-ground biomass, root mass and root mass ratio (RMR; ratio of root dry mass to total dry mass) than native *E. texanum* (Fig. 2; above-ground, *F*_1,22_ = 7.96, *P* = 0.010; root mass, *F*_1,22_ = 5.63, *P* = 0.034; and root mass ratio, *F*_1,22_ = 4.00, *P* = 0.067). We assumed consistent scaling of leaf conductance to transpiration in order to use porometery measurements to determine integrated water loss, showing that leaves of *E. cicutarium* lost 37% less water over the day of measurements than *E. texanum* (Fig. 3). This observed difference was significantly larger than that for permuted data (observed difference = 1300.18 mmol/m^2^/s, 95% confidence limits = −1196, 1155), indicating that *E. cicutarium* had significantly less leaf-level water loss than *E. texanum*. In this common environment, the total biomass of *E. texanum* was 38% less than *E. cicutarium*, so these leaf-level water loss patterns suggest greater whole-plant water-use efficiency for biomass production in the invasive species, yet potentially equivalent total water extraction from the soil (using the most conservative estimate of total biomass rather than above-ground mass and without knowing the details of canopy water use).

**Figure 4:**
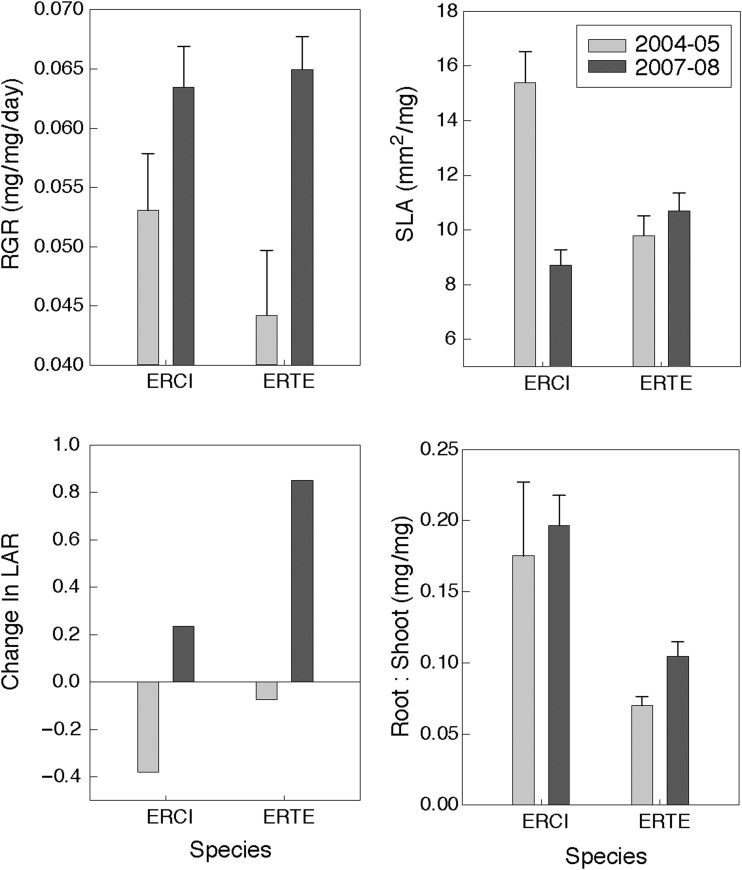
Relative growth rate (RGR; measured as the slope of ln-transformed biomass by age), specific leaf area (SLA), change in leaf area ratio (LAR_95 days after germination_ – LAR_53 days after germination_/LAR_53 days after germination_) and the ratio of root biomass to shoot biomass for both species in 2 years. Values are means ± 1 SEM. Standard errors are not given for the change in LAR, because that value was calculated from mean values at two different time points.

*Erodium cicutarium* growing in the field had faster RGR than *E. texanum* in 2004–05, but in 2007–08 their values were similar (Table 1 and Fig. 4). The RGR of both species was higher in the relatively cooler 2007–08 growing season than in the relatively warmer 2004–05 growing season (temperature during the period of intensive rainfall differed between year; see Fig. S2). The cooler year was also characterized by a lower overall density of winter annual plants (average of 376 individuals/m^2^ in 2007–08 compared with 660 individuals/m^2^ in 2004–05; http://www.eebweb.arizona.edu/faculty/venable/LTREB/). The increase in ln-transformed dry mass per unit leaf area (NAR) was greater for *E. cicutarium* than *E. texanum* in the warmer year, but there was no difference between the two species in the cooler year (resulting in a species-by-year interaction; Table 1 and Table S1). There was also a significant species-by-year interaction for SLA, such that *E. cicutarium* had significantly higher SLA than *E. texanum* in the warmer year, but not during the cooler year, when the SLA of *E. cicutarium* was closer to that of *E. texanum* (Fig. 4 and Table 1). It appears that RGR was related to differences in leaf growth, such that *E. cicutarium* in 2004–05 had significantly greater LAR and SLA than *E. texanum* (Table S2). Analysis of LAR also indicated a species-by-year interaction, such that the LAR of *E. cicutarium* was greater than *E. texanum* in the warmer year and less than *E. texanum* in the cooler year, while LAR of *E. texanum* did not differ between years (Table 1 and Table S2). Both species showed decreasing LAR during the warm year of 2004–05 and increasing LAR in response to a mid-season rain in the cool year of 2007–08 (Fig. 4). Root-to-shoot ratios were higher for *E. cicutarium* than for *E. texanum*, and greater in the cooler year than in the warmer year (Table 1 and Fig. 4). The LMR did not differ significantly depending on the species, but was greater in the warmer year than the cooler year (Table 1 and Table S2). Leaf nitrogen and Δ did not show significant differences between species or between years (Table 2 and Table S2).

**Table 1:** Results from mixed-model ANOVAs on growth components of *Erodium cicutarium* and *Erodium texanum* collected from sequential harvests of plants growing in the field during two separate growing seasons

Variable	Effect	Numerator d.f.	Denominator d.f.	*F*	*P*-value
RGR	Age	1	278	838.39	<0.0001
	Species	1	278	5.23	0.023
	Year	1	74.6	32.17	<0.0001
	Species × year	1	278	1.15	0.284
NAR	Age	1	293	81.81	<0.0001
	Species	1	293	4.71	0.031
	Year	1	293	270.8	<0.0001
	Species × year	1	293	7.91	0.005
SLA	Species	1	46	4.52	0.039
	Year	1	46	11.07	0.002
	Species × year	1	46	17.66	0.000
LAR	Species	1	46	4.46	0.040
	Year	1	46	24.9	<0.0001
	Species × year	1	46	19.02	<0.0001
Root:shoot	Species	1	46	26.2	<0.0001
	Year	1	46	6.87	0.012
	Species × year	1	46	0.18	0.677
LMR	Species	1	46	0.3	0.584
	Year	1	46	8.93	0.005
	Species × year	1	46	0.44	0.510

Plot was included as a random factor in all analyses. Relative growth rate (RGR) was analysed as the change in ln-transformed dry biomass over time, while net assimilation rate (NAR) was the change in ln-transformed mass per leaf area through time. Mid-season (95 days after germination) values of specific leaf area (SLA), leaf area ratio (LAR), root-to-shoot ratio and leaf mass ration (LMR) were ln-transformed prior to analysis.

**Table 2:** Results from ANOVAs testing whether leaf nitrogen and leaf ^13^C isotope discrimination (Δ, per mil) varied depending on species, year or the species-by-year interaction

Variable	Source of variation	d.f.	SS	MS	*F*	*P*-value
Leaf nitrogen	Species	1	0.238	0.238	0.720	0.408
	Year	1	0.002	0.002	0.000	0.946
	Year × species	1	0.581	0.581	1.760	0.204
	Error	16	5.289	0.331		
Δ	Species	1	1.470	1.470	2.830	0.113
	Year	1	0.000	0.000	0.000	0.977
	Year × species	1	0.022	0.022	0.040	0.841
	Error	15	7.800	0.520

### Competition

**Figure 5:**
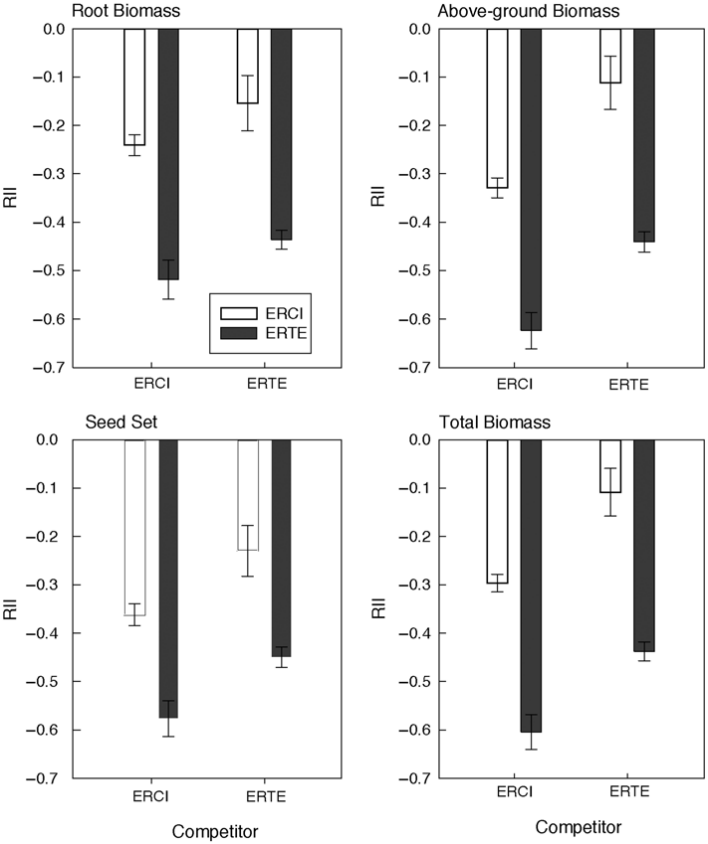
Relative interaction intensity (RII) for four response variables (root biomass, above-ground biomass, seed set and total biomass) indicating the strength of the interaction between *E. texanum* and *E. cicutarium* individuals in the competition experiment. More negative numbers indicate stronger effects of competition. See Materials and methods for the details on calculation of RII (Armas *et al.*, 2004). Error bars indicate ±1 SEM.

Both *E. cicutarium* and *E. texanum* had negative RII values for seed set, total mass, above-ground mass and root mass, indicating that both species experienced competition. The strength of the competitive interaction depended on both the identity of the focal species and the competitor (Fig. 5 and Table 3). Native *E. texanum* had lower RII values, indicating that it was more impacted by competition than non-native *E. cicutarium*. In addition, both species were more impacted by competition (more negative RII values) when grown with *E. cicutarium* than with *E. texanum* (Fig. 2 and Table 3). This pattern was consistent for all measurement variables (number of seeds produced, total biomass, above-ground mass and root biomass; Fig. 5 and Table 3).

**Table 3:** Results of two-way ANOVAs on the relative interaction intensity (RII) calculated for number of seeds, total biomass, above-ground mass or root mass to determine whether the strength of the interaction depended on the species (*E. cicutarium* or *E. texanum*), the competitor (none, *E. cicutarium* or *E. texanum*) or the interaction between species and competitor

RII variable	Source of variation	d.f.	SS	MS	*F*	*P*-value
Number of seeds	Model	3	1.59	0.53	11.33	<0.0001
	Species	1	1.57	1.57	33.72	<0.0001
	Competitor	1	0.56	0.56	11.95	0.0006
	Species × competitor	1	0.00	0.00	0.01	0.9419
	Error	234	10.92	0.05		
	Total	237.00	12.51			
Total biomass	Model	3	3.38	1.13	31.84	<0.0001
	Species	1	3.30	3.30	93.23	<0.0001
	Competitor	1	1.02	1.02	28.72	<0.0001
	Species × competitor	1	0.00	0.00	0.09	0.7584
	Error	233	8.25	0.04		
	Total	236	11.63			
Above-ground mass	Model	3	3.18	1.06	25.31	<0.0001
	Species	1	3.17	3.17	75.72	<0.0001
	Competitor	1	1.30	1.30	31.14	<0.0001
	Species × competitor	1	0.01	0.01	0.24	0.6282
	Error	233	9.76	0.04		
	Total	236	12.94			
Root mass	Model	3	3.19	1.06	24.76	<0.0001
	Species	1	2.96	2.96	68.86	<0.0001
	Competitor	1	0.23	0.23	5.43	0.0207
	Species × competitor	1	0.00	0.00	0	0.9491
	Error	233	10.00	0.04		
	Total	236	13.19

## Discussion

Identification of the mechanisms underlying differential performance of these native and invasive species across years and conditions in our system provides some insight into the challenge of understanding species invasions, particularly because we know the traits that are responsible for multi-decade population and community dynamics in this variable environment (Huxman *et al.*, 2013). It is clear that a novel combination of leaf-level physiological and whole-plant allocation strategies in comparison to the native flora contributes to the invasive success of *E. cicutarium* in the Sonoran Desert. The ability of *E. cicutarium* to achieve both high leaf-level water-use efficiency and high growth rates is consistent with previous studies suggesting that non-native species exhibited unique trait combinations that enhanced their capacity to outcompete natives in their introduced range (Blumenthal, 2006; Leishman *et al.*, 2007; Molina-Montenegro *et al.*, 2012; Kimball *et al.*, 2013). Unique trait combinations of non-native species may be expressed in the native range or may have evolved in the novel range, perhaps due to enemy release (Thebaud and Simberloff, 2001; Blair and Wolfe, 2004; Bossdorf *et al.*, 2005). Here, although we did not find support for enemy release, we did find evidence of superior competitive abilities of non-native *E. cicutarium*. The faster growth of *E. cicutarium*, along with its success in competition trials, supports numerous other studies in which invasive species exhibited higher growth rates than natives, giving them a competitive advantage (Pattison *et al.*, 1998; Daehler, 2003; Leger and Rice, 2003).

The finding that *E. cicutarium* had greater SLA and LAR in 2004–05 suggests greater ability to take advantage of warm and wet periods with high plant density when the growth rate of *E. texanum* is restricted. This ability of *E. cicutarium* is consistent with performance patterns we identify in members of the native flora with high RGR and low WUE, in which some species use canopy display as a means to increase growth rate when resources are available, termed ‘morphological responders’ by Angert *et al.* (2007). At the same time, the strategy in *E. texanum* that allows for relatively greater performance during the cool periods immediately following winter rains, ‘physiological responders’, is still operational in *E. cicutarium*, as has been seen in the photosynthetic patterns of a number of species in the flora (Huxman *et al.*, 2008). Thus, it appears that the invasive species in this system employs characteristics from both ends of the spectrum of native species' strategies for dealing with environmental variation, relying on a morphological response when resources are abundant and the physiological capacity to use soil water when it is restricted to cool periods or low amounts.

Levels of herbivory did not differ between native *E. texanum* and non-native *E. cicutarium* as would be predicted by the ERH, suggesting that *E. cicutarium* may not be experiencing a release from natural enemies (Keane and Crawley, 2002). We tested the ERH in only 1 year, and other studies of ERH have reported different results in different years, particularly in variable environments (Davis *et al.*, 2000; Agrawal *et al.*, 2005), but we do not think that this is a problem because we have not previously noticed significant levels of herbivory on *E. cicutarium*, despite 30 years of long-term demographic studies (Venable, 2007). One other, perhaps more serious, problem with our test of the ERH is that we excluded only animal herbivores and did not test for other kinds of enemies, such as fungal and viral pathogens (Mitchell and Power, 2003). However, we did not notice mortality due to pathogens for any of the annuals in our long-term study plots during the year of this study. There is a fungus that attacks *E. cicutarium* (Inouye, 1981), and seed herbivory by rodents influences both *Erodium* species (Inouye *et al.*, 1980), but these enemies influence both native and non-native species and do not support enemy release. The ERH may better explain success of non-native species in high-resource environments, where enemies are more likely to limit growth of natives (Blumenthal, 2005, 2006).

Our result that non-native *E. cicutarium* was able to achieve greater overall biomass than native *E. texanum*, despite its lower stomatal conductance, is consistent with the hypothesis that non-natives have higher resource-use efficiency (Funk and Vitousek, 2007). Other studies comparing physiological traits of natives and co-occurring invasive species have identified several common trait differences of invasive species, including higher SLA, foliar nutrients, carbon assimilation rates and growth rates (Baruch and Goldstein, 1999; Funk and Vitousek, 2007; Leishman *et al.*, 2007). We found no differences between leaf nitrogen values from field-grown plants in two different years. Non-native *E. cicutarium* had higher RGR than native *E. texanum* in a warm year in the field, which occurred in part through greater SLA and LAR. The native species also had slightly lower integrated WUE in that year, although this difference was not significant. Both of the species contrasted here have relatively high long-term WUE relative to other winter annual species in the community (Angert *et al.*, 2007). Our data suggest that they achieve this high WUE in slightly different ways. In a separate experiment, both species had higher carbon assimilation rates and a greater ability to photosynthesize at cooler temperatures relative to other winter annuals (Gremer *et al.*, 2012). In the present study, the native *E. texanum* was able to respond to a mid-season rain event in the cool year by increasing LAR more than *E. cicutarium*, perhaps due to its ability to achieve maximal photosynthetic rate at a slightly lower temperature (Gremer *et al.*, 2012). Non-native *E. cicutarium* exhibited significantly lower stomatal conductance than *E. texanum*, indicating that it is achieving high leaf-level WUE by reducing water loss in addition to investing in carbon assimilation. It appears that, in this system, the ability of non-native species to use resources efficiently does not come at the cost of a reduced ability to grow rapidly when resources are abundant, which is present in the patterns of the remaining species in the community.

There is some disagreement as to whether invasive species differ in their resource-use efficiency (Funk and Vitousek, 2007) or simply tend to have traits that allow for fast growth in high-resource environments (Daehler, 2003; Leishman *et al.*, 2007, 2010). In our study system, the two non-natives (*E. cicutarium* and *S. barbatus*) seemed to be more efficient in the way that they used resources (water) and able to achieve higher RGR when resources were abundant (Fig. S1). This may be a pattern that is general to all invasive species or it may be a result of these two strategies being so important in the dynamics of Sonoran Desert winter annual plants, given that this system is one of the few in existence where the physiological and growth strategies for the dominant species is well documented in the context of decades of generations of population dynamics (Huxman *et al.*, 2013). Our data suggest that invasive success derives from the combination of these water-use efficiency and growth response strategies in the context of the relative values existing in the local flora rather than the ranking values of either trait syndrome in isolation.

Results from our competition experiment indicated that non-native *E. cicutarium* was less impacted by competition than *E. texanum*. Faster RGR of *E. cicutarium* may be a factor, because size has been demonstrated to go along with competitive ability in other studies (Goldberg and Fleetwood, 1987; Gurevitch *et al.*, 1990; Wang *et al.*, 2010; Gremer *et al.*, 2013). Invasive species frequently outcompete natives in high-resource conditions similar to those in our greenhouse experiment (Daehler, 2003; Corbin and D'Antonio, 2004). It is unclear whether *E. cicutarium* would outcompete *E. texanum* in low-resource conditions as well, but our long-term demographic data indicate that *E. cicutarium* has maintained higher fitness than other winter annual species during dry years (Kimball *et al.*, 2012). From our measurements of growth in two different years, we would predict that non-native *E. cicutarium* would outcompete native *E. texanum* in the field, especially in warm years. Native *E. texanum* may exhibit greater growth in cool years than in warm years, yet this would probably not allow the native species to outcompete *E. cicutarium*.

Among-species trade-offs are thought to be important in maintaining diversity in communities (Kneitel and Chase, 2004; Agrawal *et al.*, 2010). It is likely that the ability of *E. cicutarium* to grow quickly while simultaneously controlling leaf-level water loss will contribute to instability of the Sonoran Desert winter annual community (Molina-Montenegro *et al.*, 2012; Kimball *et al.*, 2013). There has been a warming and drying of the Sonoran Desert, which has been accompanied by a delayed arrival of germination-triggering rain events, favouring species that germinate at cooler temperatures (Kimball *et al.*, 2010). The increase in abundance of *E. cicutarium*, along with other species at the high-WUE end of the trade-off axis, seems to be due partly to the ability of high-WUE species to germinate in these cooler conditions and partly to their ability to withstand years with high late-season temperatures (Kimball *et al.*, 2010, 2012; Gremer *et al.*, 2012). This pattern of changing weather conditions suggests that *E. cicutarium* may continue to increase in abundance, probably due to its superior physiological traits.

## Supplementary material

Supplementary material is available at *Conservation Physiology* online.

Supplementary Data
