# Supplementary material for: High water-use efficiency and growth contribute to success of non-native Erodium cicutarium in a Sonoran Desert winter annual community
Source: Conserv Physiol. 2014 Mar 5;2(1):cou006. doi: 10.1093/conphys/cou006 (PMC4806723; doi:10.1093/conphys/cou006)
Supplement: Supplementary Data [file supp_2_1_cou006__index.html]

High water-use efficiency and growth contribute to success of non-native Erodium cicutarium in a Sonoran Desert winter annual community — Supplementary Data 

# High water-use efficiency and growth contribute to success of non-native *Erodium cicutarium* in a Sonoran Desert winter annual community

## Supplementary Data

Supplementary Data

**Files in this Data Supplement:**

- Supplementary Data - Docx file
