# Supplementary material for: High water-use efficiency and growth contribute to success of non-native Erodium cicutarium in a Sonoran Desert winter annual community
Source: Conserv Physiol. 2014 Mar 5;2(1):cou006. doi: 10.1093/conphys/cou006 (PMC4806723; doi:10.1093/conphys/cou006)
Supplement: Supplementary Data [file supp_cou006_cou006supp.docx]

Supplementary Material

**Figures**


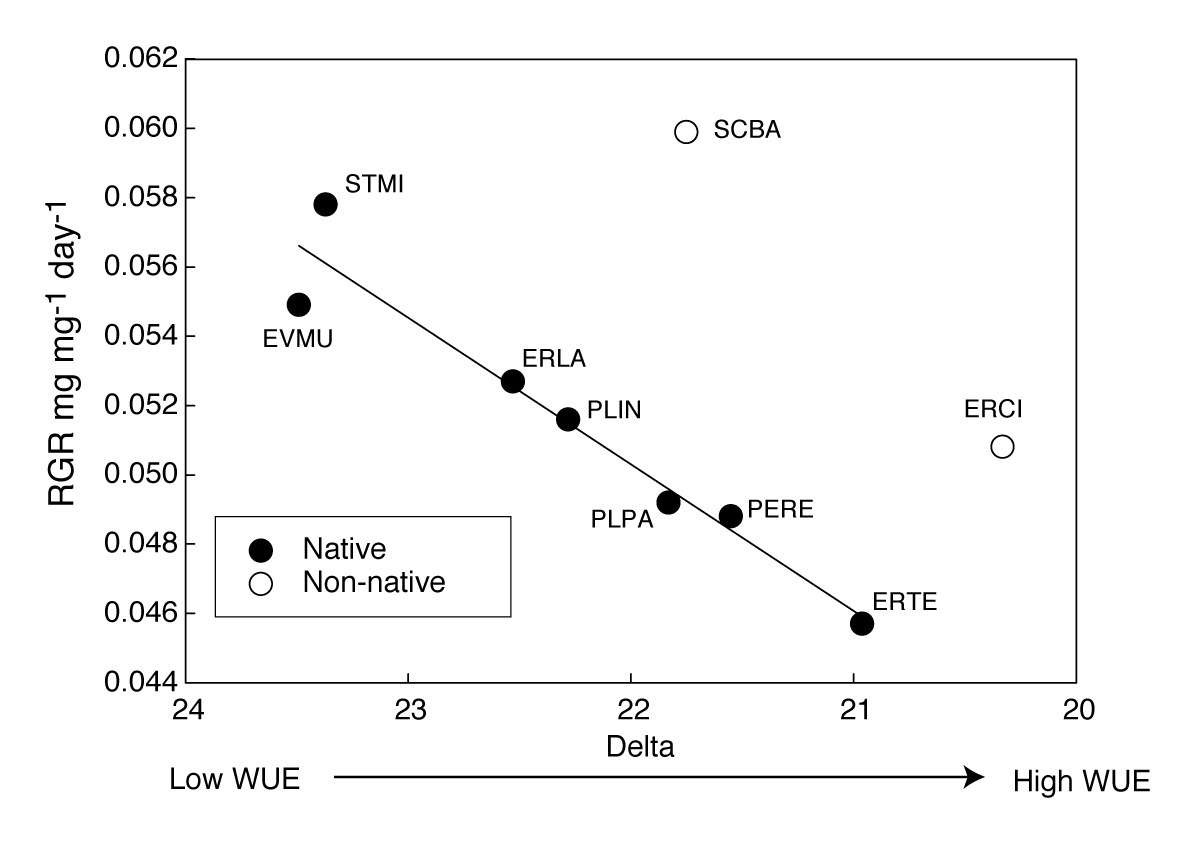


Figure S1: Carbon isotope discrimination (Δ) is an indicator that decreases with higher integrated water-use efficiency (WUE, redrawn from Angert *et al.* 2009). ItWUE is strongly related to relative growth rate (RGR) consistent with a tradeoff between growth rate and WUE. Non-native species (open circles) have greater RGR for any given WUE than native species (closed circles). STMI=*Stylocline micropoides*, EVMU=*Evax multicaulis*, ERLA=*Eriophyllum lanosum*, PLIN=*Plantago insularis*, PLPA=*Plantago patagonica*, PERE=*Pectocarya recurvata*, ERTE=*Erodium texanum*, SCBA=*Schismus barbatus*, and ERCI=*Erodium cicutarium*.


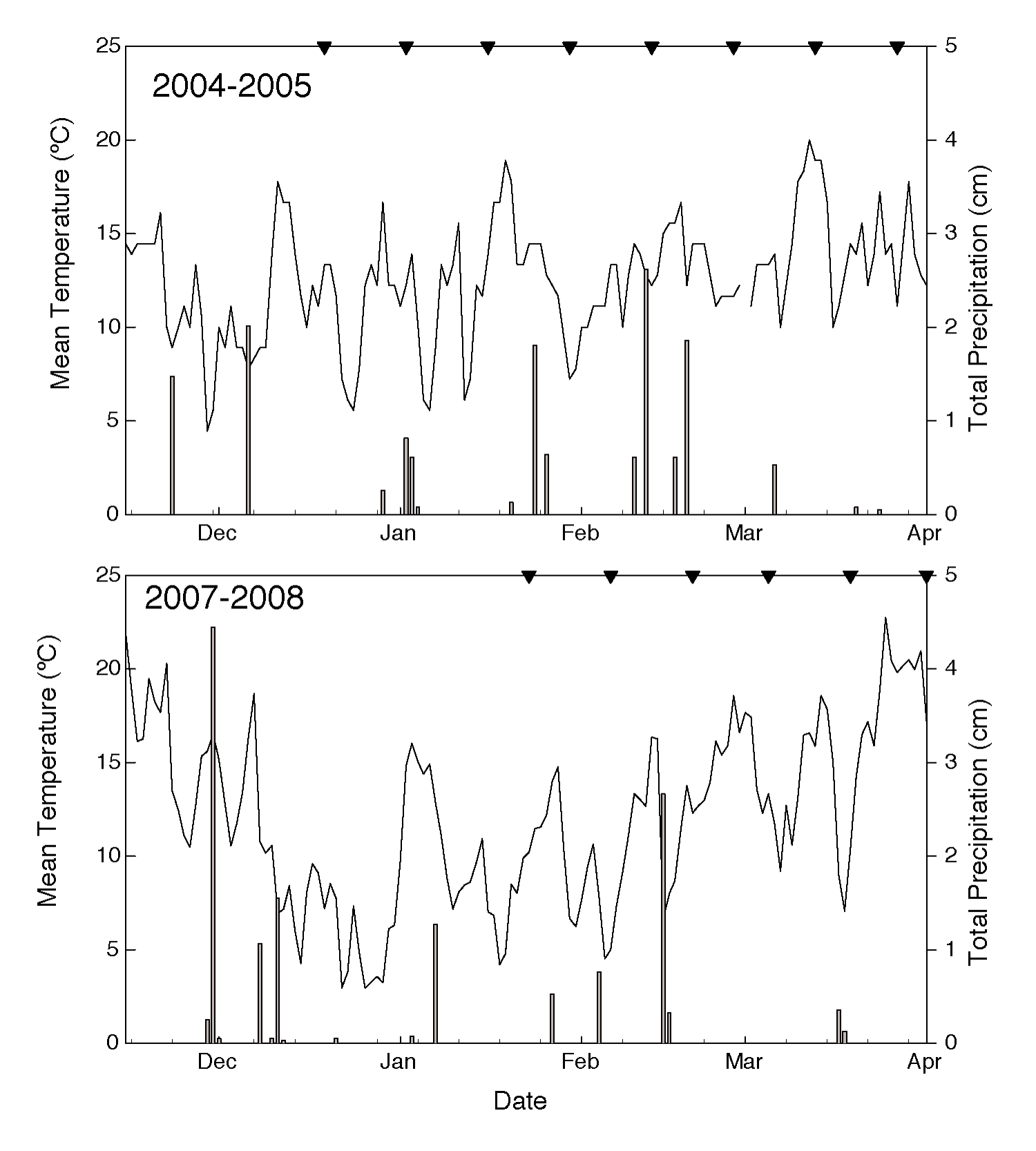


Figure S2. Temperature (solid lines) and precipitation (bars) during the 2004-05 and 2007-08 growing seasons. Triangles at the top of the graphs indicate dates of sequential harvests. Harvesting began later in 2007-08 despite similar timing of germination, so the first two harvests from 2004-05 were eliminated from the analyses.

Table S1. Mean growth, leaf N, and Δ values of *Erodium cicutarium* and *Erodium texanum* collected during the 2004-05 and the 2007-08 growing seasons. RGR and NAR were measured as the linear relationship of ln-transformed mass and mass per leaf area over time. Other mean trait values are from plants harvested 95 days after germination.

| Species | Year | Trait | Mean | SE |
| --- | --- | --- | --- | --- |
| ERCI | 2004-05 | RGR | 0.0531 | 0.0048 |
| ERTE | 2004-05 | RGR | 0.0442 | 0.0055 |
| ERCI | 2007-08 | RGR | 0.0635 | 0.0034 |
| ERTE | 2007-08 | RGR | 0.0650 | 0.0028 |
| ERCI | 2004-05 | NAR | 0.0116 | 0.0019 |
| ERTE | 2004-05 | NAR | 0.0078 | 0.0029 |
| ERCI | 2007-08 | NAR | 0.0114 | 0.0018 |
| ERTE | 2007-08 | NAR | 0.0106 | 0.0026 |
| ERCI | 2004-05 | N | 4.30 | 0.1684 |
| ERTE | 2004-05 | N | 3.74 | 0.3985 |
| ERCI | 2007-08 | N | 3.94 | 0.1082 |
| ERTE | 2007-08 | N | 4.07 | 0.2560 |
| ERCI | 2004-05 | Δ | 20.33 | 0.2470 |
| ERTE | 2004-05 | Δ | 20.96 | 0.4477 |
| ERCI | 2007-08 | Δ | 20.41 | 0.2840 |
| ERTE | 2007-08 | Δ | 20.90 | 0.2528 |
| ERCI | 2004-05 | SLA | 15.40 | 1.1280 |
| ERTE | 2004-05 | SLA | 9.79 | 0.7247 |
| ERCI | 2007-08 | SLA | 8.72 | 0.5595 |
| ERTE | 2007-08 | SLA | 10.70 | 0.6621 |
| ERCI | 2004-05 | Root:Shoot | 0.175 | 0.0520 |
| ERTE | 2004-05 | Root:Shoot | 0.070 | 0.0066 |
| ERCI | 2007-08 | Root:Shoot | 0.196 | 0.0212 |
| ERTE | 2007-08 | Root:Shoot | 0.105 | 0.0100 |
| ERCI | 2004-05 | LAR | 18.10 | 4.7231 |
| ERTE | 2004-05 | LAR | 8.57 | 0.4865 |
| ERCI | 2007-08 | LAR | 6.67 | 0.4101 |
| ERTE | 2007-08 | LAR | 8.25 | 0.5634 |
| ERCI | 2004-05 | LMR | 0.849 | 0.0339 |
| ERTE | 2004-05 | LMR | 0.889 | 0.0289 |
| ERCI | 2007-08 | LMR | 0.769 | 0.0178 |
| ERTE | 2007-08 | LMR | 0.774 | 0.0324 |
